# Supplementary material for: Neuronal processing of facial expressions in the primate superior colliculus
Source: bioRxiv. 2026 Sep 12:2026.09.10.750132. Preprint. [Version 1] doi: 10.64898/2026.09.10.750132 (PMC13622660; doi:10.64898/2026.09.10.750132)
Supplement: Supplementary file 1 [file NIHPP2026.09.10.750132V1-supplement-1.pdf]

bioRxiv preprint doi: https://doi.org/10.64898/2026.09.10.750132; this version posted September 12, 2026. The copyright holder for this preprint (which was not certified by peer review) is the author/funder. This article is a US Government work. It is not subject to copyright under 17 USC 105 and is also made available for use under a CC0 license.

Supplementary Figures

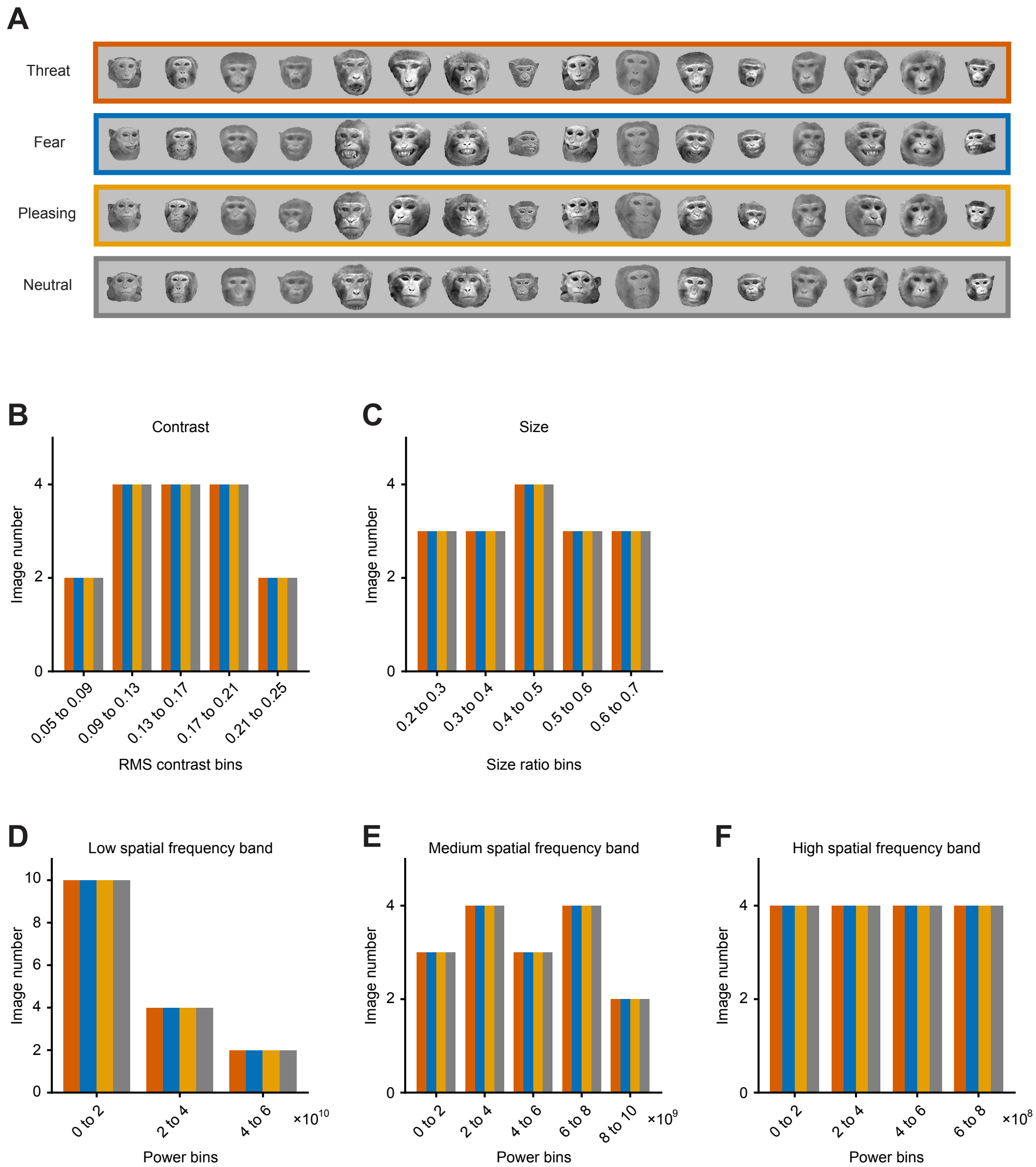

Fig. S1 | Distributions of low-level visual features were matched across object categories. (A) The facial expression image set consisted of 64 images, 16 each from four facial expression categories (threat, fear, pleasing and neutral). Across the four facial expressions, the distribution of five low-level features was matched. The features are: (B) RMS contrast; (C) Size ratio (number of object pixels divided by number of

bioRxiv preprint doi: https://doi.org/10.64898/2026.09.10.750132; this version posted September 12, 2026. The copyright holder for this preprint (which was not certified by peer review) is the author/funder. This article is a US Government work. It is not subject to copyright under 17 USC 105 and is also made available for use under a CC0 license.

pixels in the 6 deg aperture); (D) Spectral power in a low spatial frequency (SF) band (0.1667 to 0.6525 cycles/degree); (E) Spectral power in an intermediate SF band (0.6525 to 2.5544 cycles/degree); (F) Spectral power in a high SF band (2.5544 to 10 cycles/degree). Distribution matching was confirmed with the Kolmogorov–Smirnov test ($p > 0.05$, for each of the possible pairwise comparisons of object categories for each low-level feature). Additionally, mean luminance of all object images was matched to the mean luminance of the background ($32.6 cd/m^2$).

bioRxiv preprint doi: https://doi.org/10.64898/2026.09.10.750132; this version posted September 12, 2026. The copyright holder for this preprint (which was not certified by peer review) is the author/funder. This article is a US Government work. It is not subject to copyright under 17 USC 105 and is also made available for use under a CC0 license.

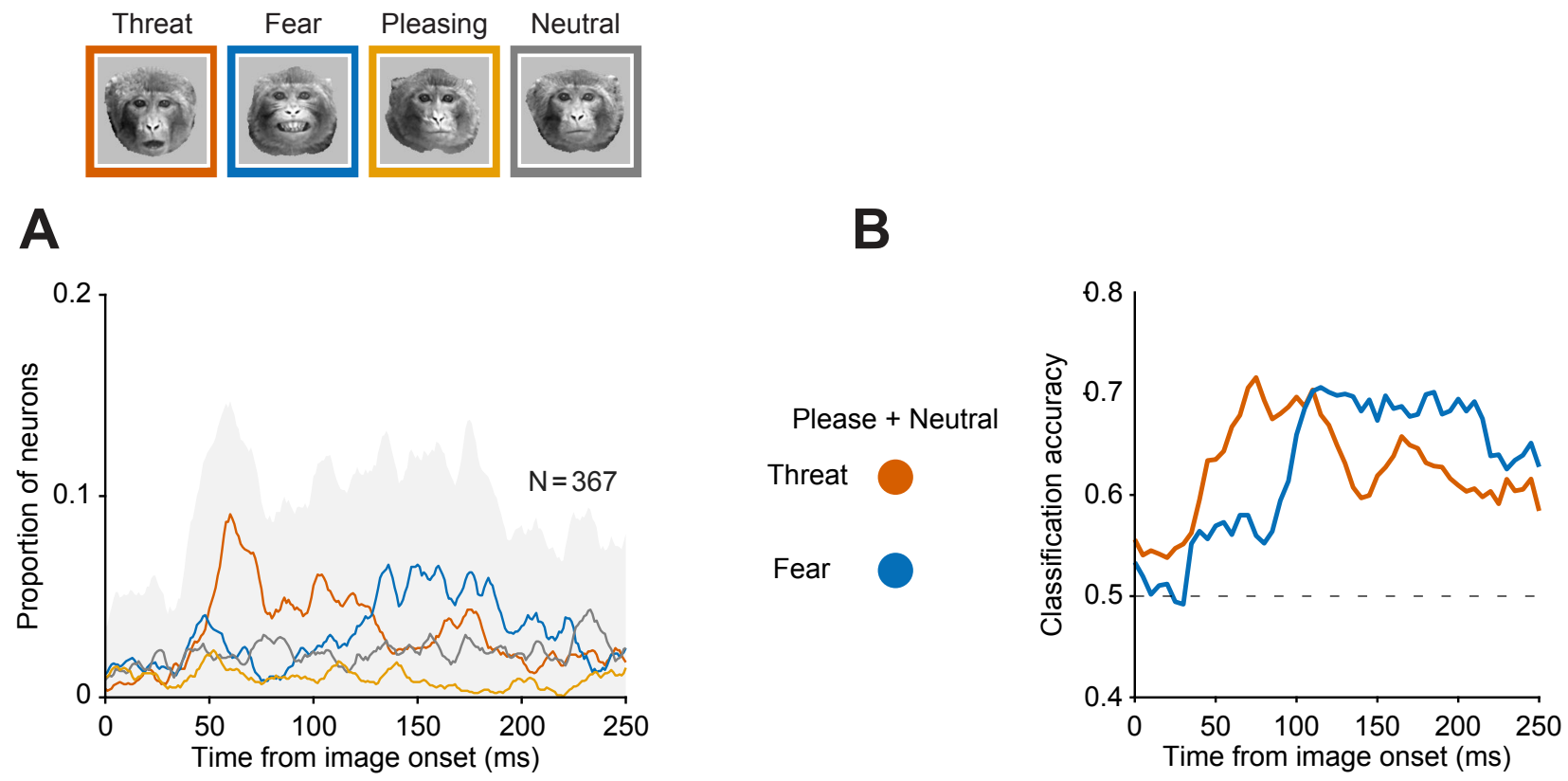

Fig. S2 | Facial expression preferences in SC persists after regressing out low-level visual features. (A) Proportion of SC neurons exhibiting facial-expression preferences over time after removing the contribution of low-level visual features. For each neuron and time bin, responses were modeled using a multilinear regression with five image features as regressors: RMS contrast, size ratio, and power in low, medium, and high spatial-frequency bands. Facial-expression preference was then assessed on the residual responses using a Kruskal–Wallis H test with facial expression as the group variable ($p < 0.05$). Gray shaded area indicates the overall proportion of expression-modulated neurons (preference for any facial expression). Individual colored curves denote the proportion of neurons displaying a preference for a specific facial expression. (B) Cross-validated decoding accuracy computed from the residual responses. Linear classifiers were trained to discriminate threat faces from nonnegative expressions and fear faces from nonnegative expressions, where nonnegative expressions included pleasing and neutral faces.

bioRxiv preprint doi: https://doi.org/10.64898/2026.09.10.750132; this version posted September 12, 2026. The copyright holder for this preprint (which was not certified by peer review) is the author/funder. This article is a US Government work. It is not subject to copyright under 17 USC 105 and is also made available for use under a CC0 license.

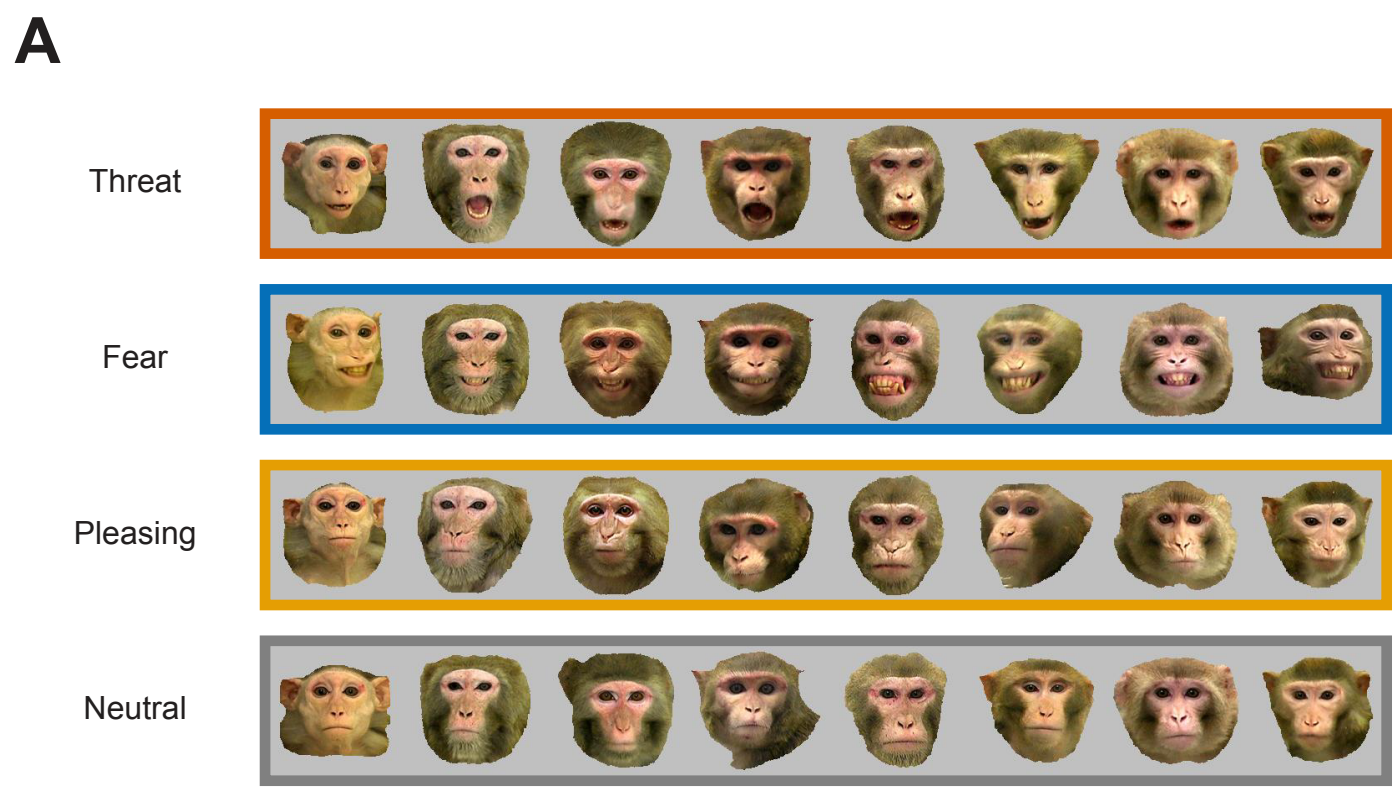

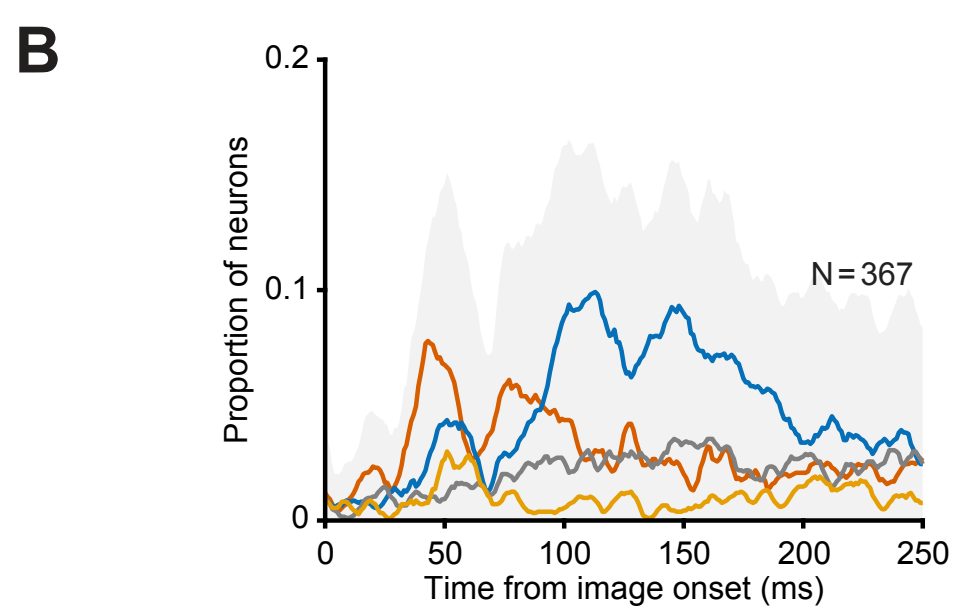

Fig. S3 | Early threat- and late fear-related pattern was preserved for original color images. (A) Original color facial-expression image set previously shown to elicit expression-selective neural activity in the macaque amygdala. (B) Time course of the proportion of neurons showing significant color-facial-expression preference. Gray shaded area indicates the overall proportion of expression-modulated neurons (preference for any facial expression). Individual colored curves denote the proportion of neurons displaying a preference for a specific facial expression.

bioRxiv preprint doi: https://doi.org/10.64898/2026.09.10.750132; this version posted September 12, 2026. The copyright holder for this preprint (which was not certified by peer review) is the author/funder. This article is a US Government work. It is not subject to copyright under 17 USC 105 and is also made available for use under a CC0 license.

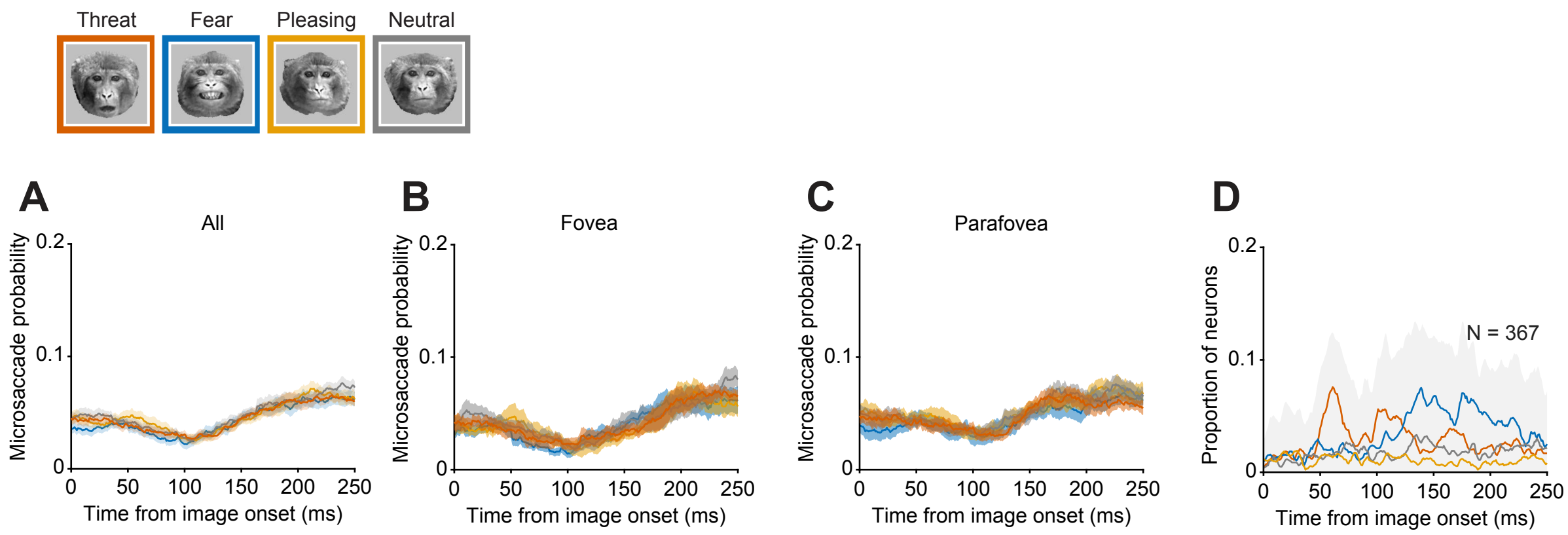

Fig. S4 | Facial expression preferences in SC cannot be explained by the occurrence of microsaccades. Probability of microsaccades over time in 20ms non-overlapping bins aligned to image onset for the four facial expressions, shown for all trials (A), foveal conditions (B), and parafoveal conditions (C). There was no significant difference in microsaccade probability across facial expressions in any time bin (Kruskal–Wallis H test with facial expression as the group variable, $p > 0.05$). (D) Proportion of SC neurons exhibiting facial-expression preferences (Kruskal–Wallis H test with facial expression as the group variable, $p < 0.05$) after excluding trials containing microsaccades from 0 to 250 ms after image onset. Gray shaded area indicates the overall proportion of expression-modulated neurons (preference for any facial expression). Individual colored curves denote the proportion of neurons displaying a preference for a specific facial expression.

bioRxiv preprint doi: https://doi.org/10.64898/2026.09.10.750132; this version posted September 12, 2026. The copyright holder for this preprint (which was not certified by peer review) is the author/funder. This article is a US Government work. It is not subject to copyright under 17 USC 105 and is also made available for use under a CC0 license.

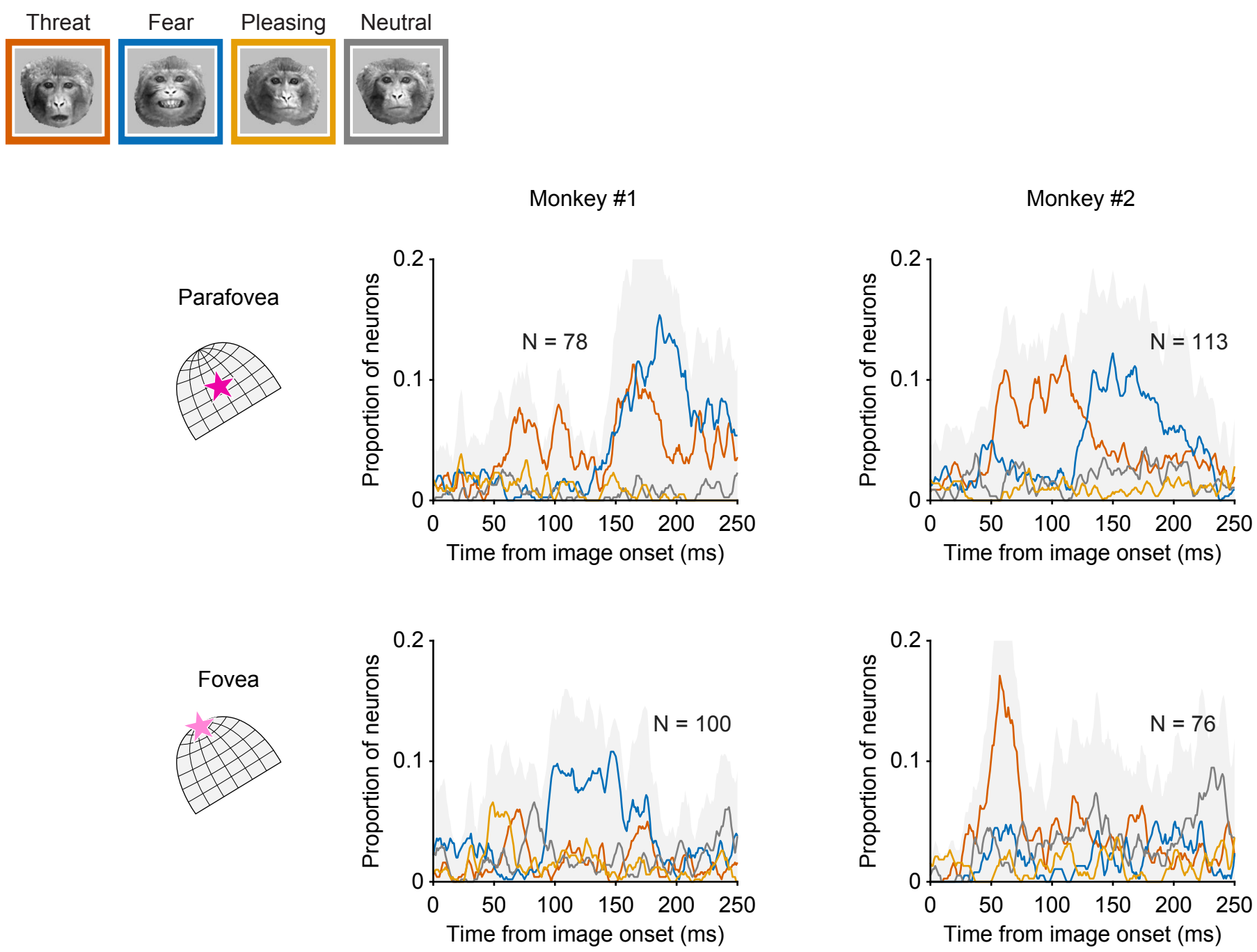

Fig. S5 | Expression-related signals in individual animals. Time courses show the proportion of neurons with significant facial-expression modulation, plotted separately for monkey 1 and monkey 2. Gray shading indicates the overall proportion of expression-modulated neurons, defined as neurons with a significant effect of facial expression regardless of preferred expression. Colored curves indicate the proportion of neurons preferring each facial expression. Top row shows parafoveal visually responsive neurons and the bottom row shows foveal neurons. Across animals, the most robust shared pattern was found in parafoveal SC, where threat-related modulation emerged early and persisted, whereas fear-related modulation emerged later. Foveal expression-related signals showed individual-specific differences in the relative strength of threat- and fear-related information.
